# Exon Organization and Novel Alternative Splicing of *Ank3* in Mouse Heart

**DOI:** 10.1371/journal.pone.0128177

**Published:** 2015-05-29

**Authors:** Gokay Yamankurt, Henry C. Wu, Michael McCarthy, Shane R. Cunha

**Affiliations:** 1 Department of Chemistry, Northwestern University, Evanston, Illinois, United States of America; 2 Department of Integrative Biology and Pharmacology, UT Health, Houston, Texas, United States of America; International Centre for Genetic Engineering and Biotechnology, ITALY

## Abstract

Ankyrin-G is an adaptor protein that links membrane proteins to the underlying cytoskeletal network. Alternative splicing of the *Ank3* gene gives rise to multiple ankyrin-G isoforms in numerous tissues. To date, only one ankyrin-G isoform has been characterized in heart and transcriptional regulation of the *Ank3* gene is completely unknown. In this study, we describe the first comprehensive analysis of *Ank3* expression in heart. Using a PCR-based screen of cardiac mRNA transcripts, we identify two new exons and 28 alternative splice variants of the *Ank3* gene. We measure the relative expression of each splice variant using quantitative real-time PCR and exon-exon boundary spanning primers that specifically amplify individual *Ank3* variants. Six variants are rarely expressed (<1%), while the remaining variants display similar expression patterns in three hearts. Of the five first exons in the *Ank3* gene, exon 1d is only expressed in heart and skeletal muscle as it was not detected in brain, kidney, cerebellum, and lung. Immunoblot analysis reveals multiple ankyrin-G isoforms in heart, and two ankyrin-G subpopulations are detected in adult cardiomyocytes by immunofluorescence. One population co-localizes with the voltage-gated sodium channel Na_V_1.5 at the intercalated disc, while the other population expresses at the Z-line. Two of the rare splice variants excise a portion of the ZU5 motif, which encodes the minimal spectrin-binding domain, and these variants lack β-spectrin binding. Together, these data demonstrate that *Ank3* is subject to complex splicing regulation resulting in a diverse population of ankyrin-G isoforms in heart.

## Introduction

Normal excitation-contraction coupling in skeletal and cardiac myocytes requires that the relative arrangement of integral membrane proteins remains unperturbed throughout the contraction cycle. Some of these membrane proteins facilitate structural continuity between adjacent myocytes, while other membrane proteins mediate the ionic flux that underlies excitation-contraction coupling. Adaptor proteins like ankyrin are critical for the retention and scaffolding of integral membrane proteins to the underlying cytoskeleton. By scaffolding specific membrane proteins and signaling molecules, ankyrins also contribute to the functional specialization of subcellular domains within myocytes.

Alternative splicing of an ankyrin gene produces different isoforms that display unique functions and subcellular distribution. In fact, alternative splicing of the *Ank3* gene results in numerous ankyrin-G isoforms that have been detected in various tissues including brain, skeletal muscle, lung, and kidney [1–6]. In heart, only one ankyrin-G isoform has been characterized, yet numerous membrane proteins have been shown to interact with ankyrin-G including connexin 43, β dystroglycan, and voltage-gated sodium channels [7–12]. These membrane proteins are expressed at distinct membrane domains in ventricular cardiomyocytes such as the intercalated disc, transverse(T)-tubule, and costamere [7, 8, 10–12]. Considering these findings, we hypothesize that the heart expresses more than one isoform of ankyrin-G.

This study is the first to report the comprehensive analysis of *Ank3* expression and alternative splicing in the heart. We demonstrate that the heart expresses multiple ankyrin-G isoforms and that ankyrin-G isoforms are detected at the intercalated discs and T-tubules of individually isolated cardiomyocytes. Using a PCR-based screen of cardiac mRNA, we identify two new exons in the *Ank3* gene and 28 novel splicing events in *Ank3* transcripts. We measure the relative ventricular expression of each splice junction by quantitative real-time PCR with transcript-specific primers. We demonstrate that expression of exon 1d, one of the five first *Ank3* exons, is restricted to heart and skeletal muscle. We evaluate some of the alternative splice isoforms for altered function and find that two rare isoforms of the ankyrin-G spectrin-binding domain lack spectrin binding. In summary, this study demonstrates that the *Ank3* gene is subject to complex splicing regulation resulting in numerous ankyrin-G isoforms in heart. We anticipate that these different isoforms underlie the diversity of ankyrin-G functions and subcellular distribution within cardiomyocytes.

## Materials and Methods

### RNA isolation, reverse transcription, and PCR amplification of *Ank3* transcripts

RNA was isolated from mouse tissues with GenEluteMammalian RNA kit (Sigma Aldrich). cDNA was synthesized using SuperScript III (Life Technologies). *Ank3* transcripts were amplified using nine overlapping primer sets using Phusion polymerase (Finnzymes) from mouse heart cDNA. PCR products were purified, ligated into pCR2.1-TOPO vector (Life Technologies), and sequenced.

### Quantitative RT-PCR analysis of alternative *Ank3* transcripts

Exon-exon boundary spanning primers containing ~12 base pairs from each exon were designed to PCR-amplify specific *Ank3* splice junctions as previously described [13]. cDNA was synthesized from mRNA isolated from three age- and sex-matched mice. For each primer set, quantitative rt-PCR was performed in triplicate using SYBR Green Jumpstart Taq mix (Sigma Aldrich) and experiments were repeated three times. Analysis of the data was performed using a modified version of the Pfaffl method to incorporate primer efficiencies [14]. First, alternative *Ank3* splice junctions were grouped according to shared exons (e.g. E15/16 is grouped with E15/17). The fold difference was determined using the highest threshold cycle (C_T_) value within that group as a reference. Percentage of abundance of each junction was calculated by comparing the C_T_ value of that junction to the sum of all C_T_ values for that group (e.g. the sum of E15/16 and E15/17 equal 100%). All errors were propagated through the fold difference and percentage calculations as standard deviations. The results were graphed with individual bars representing the average of a technical replicate and error bars represent standard deviations. Nucleotide sequences, amplicon lengths, annealing temperatures, and efficiencies for qt-PCR primer sets are listed in S1 Table.

### Molecular cloning of β-spectrins, ankyrin-G SBD and CTD variants

Spectrin repeats 13 to 17 of β1- and β2-spectrins were PCR-amplified from heart cDNA using primers for β1-spectrin (TCTATGTCATCTCCGATGAGATCCC, CTACAGTGACTCCCAGGAACTAGAC) and β2-spectrin (AGAGTGCTGTCTCCATGTTG, CTAAGTATCCCACTGCTGCTGGGA). PCR products were sequenced to confirm the partial cDNAs of β1- and β2-spectrins. Products were subcloned into pGEX6p-1 for bacterial over-expression. Novel ankyrin-G SBD variants were generated by ligating *Ank3* fragments PCR-amplified with primer sets 5 and 6 into pcDNA3.1. *Ank3* fragments with novel splice junctions lacking exon 31 were PCR-amplified with primers (ATGACGGAGGAAATTATGACCAC, GGAGAGAGAACTTATCGTCCTT). *Ank3* fragments PCR-amplified with primer set 6 used primers (GAACTTATCGTCCTTCGGAGC, TCATGGTGATCGGCTTATG). Novel ankyrin-G CTD variants were PCR amplified with the primer set (ATGAGGATGGCGATAGTAGCCG, AACTTCTCCCTGCTTAGGCTC) and subcloned into the lenti-viral vector pCDH1-MCS (Systems Biosciences).

### *In vitro* binding assays

GST-fusion proteins of β1- and β2-spectrins were over-expressed and purified as described [13]. *In vitro* translated (IVT) products of AnkG-SBD variants (wt, ΔE31, ΔE28-31) were prepared using TnT T7-Coupled Reticulate Lysate System (Promega) and binding assays were performed as described [13].

### Immunoblot analysis of ankyrin-G expression

Frozen mouse cerebellum and heart were pulverized with a mortar and pestle and resuspended in 4 volumes of RIPA buffer (50 mM Tris pH 8.0, 150 mM NaCl, 0.1% SDS, 0.5% sodium deoxycholate, 1% Triton X-100, 1 mM PMSF and 1X protease inhibitor cocktail (Sigma Aldrich)). Lysate was homogenized with a dounce homogenizer, incubated on ice for 30 minutes, and then centrifuged at 20,000 x g for 15 minutes at 4°C. Supernatants were collected and protein lysates were separated by SDS-PAGE and transferred to nitrocellulose membrane (GE Healthcare). Membranes were incubated with affinity-purified ankyrin-G IgG (0.5 μg/ml) or Na^+^/H^+^ exchange regulatory factor (NHERF) (Sigma Aldrich) overnight at 4°C. Immunoreactive polypeptides were visualized using SuperSignal West Pico Chemiluminescent Substrate (Pierce).

### Isolation and imaging of individual adult mouse cardiomyocytes

Adult mouse cardiomyocytes were isolated as described previously [15]. This study was carried out in strict accordance with the recommendations in the Guide for the Care and Use of Laboratory Animals of the National Institutes of Health. The protocol was approved by the Animal Welfare Committee at University of Texas Health Science Center at Houston (permit #13–083). 3 month-old mice were anesthetized with intraperitoneal injection of tribromoethanol-Avertin (Sigma T48402) at 0.2 ml/10g of 1.25% solution, euthanized by removing the heart, and all efforts were made to minimize suffering. Hearts from wild type mice were placed in ice-cold saline and the aorta was cannulated. Hearts were first perfused with warm perfusion buffer for a few minutes, followed by perfusion with digestion buffer containing collagenase (Worthington, Collagenase type II 305U/mg). Once digested, hearts were minced and triturated, then centrifuged at 300 rpm x 5 minutes at 4°C. Supernatant was removed and cells were immediately fixed by adding ice-cold 100% ethanol in excess, and kept in -20°C until use.

Cells were washed in ice-cold phosphate-buffered saline (PBS, pH 7.4) 3x. Cells were then blocked with 5% normal goat serum and 0.075% TritonX-100 for 30 minutes at room temperature then incubated in primary antibodies overnight at 4°C. Primary antibodies used were: voltage-gated sodium channel Na_V_1.5 (1:500, [16], ankyrin-G (1:50, clone N106/20, UC Davis/NIH NeuroMab Facility), α-actinin (1:200, Sigma), and ankyrin-G (1:50, [17]). Secondary antibodies used were goat anti-rabbit conjugated to Alexa Fluor 488 and goat anti-mouse conjugated to Alexa Fluor 568 (1:500, LifeTechnologies). Hoechst 33258 (1:1000, LifeTechnologies) was used for nuclear staining after removal of the secondary antibody. ProLong Gold Antifade reagent (LifeTechnologies) was used for mounting coverslips. Images were obtained with a Nikon A1 confocal microscope (Nikon, Melville, NY) equipped with 40X oil, numerical aperture 1.4 objective.

### Isolation and viral transduction of mouse neonatal cardiomyocytes

Primary cultures of cardiomyocytes were prepared from P1 wild-type neonatal mouse hearts as previously described [18]. This study was carried out in strict accordance with the recommendations in the Guide for the Care and Use of Laboratory Animals of the National Institutes of Health. The protocol was approved by the Animal Welfare Committee at University of Texas Health Science Center at Houston (permit #13–083). Neonatal mice were euthanized by decapitation and all efforts were made to minimize suffering. Briefly, hearts were enzymatically and mechanically dispersed in Ham’s F10 with 0.05% trypsin for two 15-minute incubations at 37°C followed by a 50-minute incubation in collagenase type II (0.2mg/mL, Sigma). Pelleted and dispersed myocytes were pre-plated for 4 hours at 37°C in complete growth media (40% Ham’s F-10, 40% Dulbecco’s Modified Eagle’s Media with high glucose, and 20% fetal bovine serum). Non-adherent cardiomyocytes were pelleted, re-suspended in fresh complete growth media, and plated on MatTek plates (MatTek Corporation, Ashland, MA). 48 hours later, the media was replaced with defined growth media (1 μg/mL insulin, 5μg/mL transferrin, 1nM LiCl, 1nM NaSeO_4_, 0.1nM thyroxine) to prevent overgrowth of fibroblasts. Following 3 days in culture, cardiomyocytes were transduced with lenti-viral constructs of GFP-tagged full-length or truncated AnkG-CTDs, and imaged 4 days later by confocal microscopy. Primary antibodies used were: GFP (1:500, SC-8334, Santa Cruz Antibodies), α-actinin (1:1000, Sigma), and ankyrin-G (1:500, [17]).

### Statistical Analysis

Statistical analyses were performed using Prism (GraphPad Software, Inc; version 6.0). One-way ANOVA followed by Tukey’s multiple comparison test or unpaired Student’s *t*-test was used to analyze C_T_ values from qt-PCR experiments to determine whether the expression differences in splice junctions were statistically significant.

## Results

### The heart expresses a heterogeneous population of ankyrin-G isoforms

**Fig 1:**
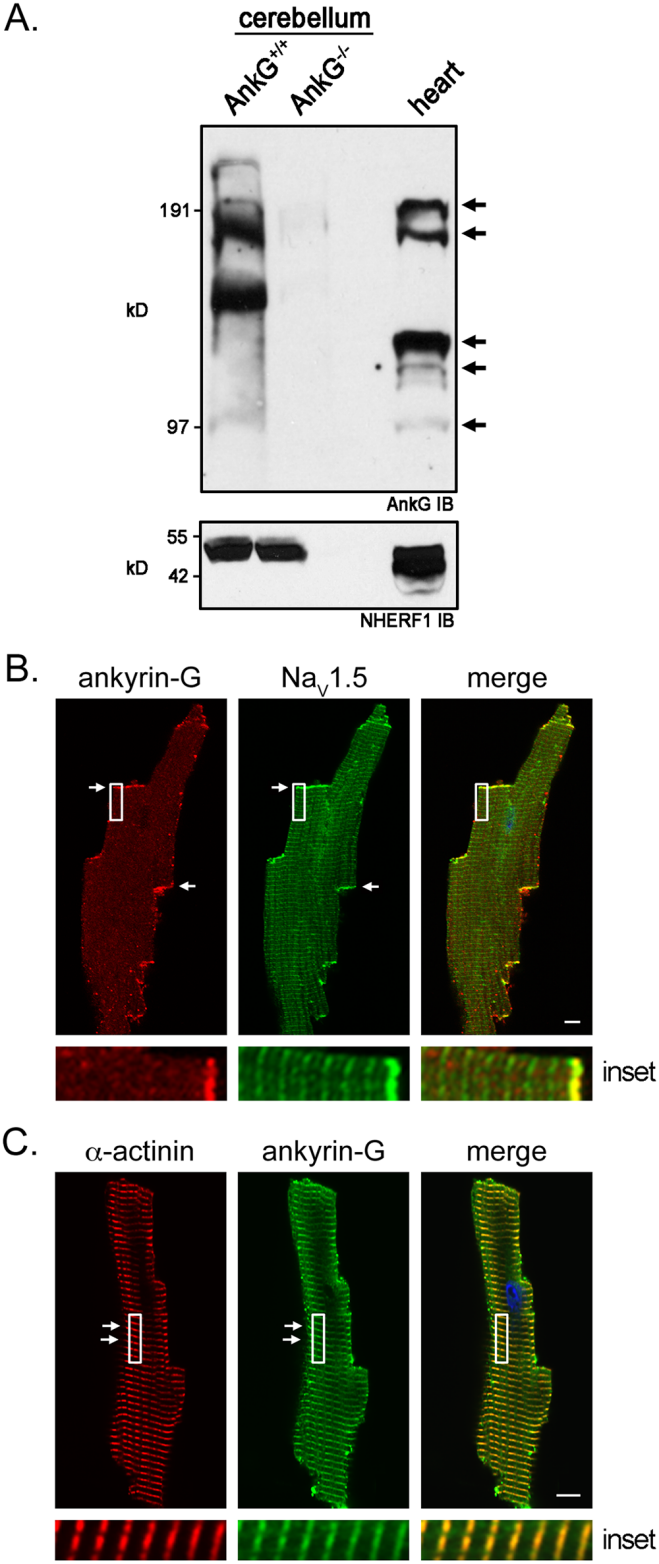
Immunoblot and immunofluorescent detection of ankyrin-G isoforms in heart. (**A**) Ankyrin-G immunoblot demonstrates expression of different isoforms in heart (arrows). Loss of ankyrin-G immunoreactive polypeptides in ankyrin-G null cerebellums demonstrates antibody specificity. NHERF1 immunoblot demonstrates similar loading of protein lysates. (**B**) Immunofluorescent co-localization of ankyrin-G (monoclonal antibody) and the voltage-gated sodium channel Na_V_1.5 at intercalated discs of an individually isolated adult cardiomyocyte (white arrows and highlighted in the inset). (**C**) Immunofluorescent co-localization of ankyrin-G (polyclonal antibody) and α-actinin at Z-lines of individually isolated adult cardiomyocytes (white arrows and highlighted in the inset). Scale bar represents 10 microns.

To determine if the heart expresses more than one ankyrin-G isoform, we performed immunoblot analysis of ankyrin-G protein expression in heart using a polyclonal antibody to ankyrin-G [10]. To confirm the specificity of the ankyrin-G antibody, we also performed immunoblot analysis of ankyrin-G expression in wild-type and ankyrin-G null cerebellums. In contrast to ankyrin-G null cerebellums, wild-type cerebellums express numerous ankyrin-G immunoreactive polypeptides (Fig 1A). Likewise, we detected multiple ankyrin-G isoforms in heart lysate from adult mice.

We performed confocal microscopy to evaluate ankyrin-G subcellular localization with resident proteins of the intercalated disc and T-tubules in cardiomyocytes isolated from adult mice. Using a monoclonal antibody to ankyrin-G, we find a population of ankyrin-G co-localizes with the voltage-gated sodium channel Na_V_1.5 at the intercalated discs (Fig 1B). The commercially available monoclonal antibody was generated against the spectrin-binding, death, and C-terminal domains of human ankyrin-G. Additionally, using a polyclonal antibody to ankyrin-G, we demonstrate another population of ankyrin-G co-localizes with α-actinin, a cytoskeletal component of the Z-line that underlies 60% of the T-tubular network (Fig 1C) [19]. The polyclonal antibody was generated against the death and C-terminal domains of human ankyrin-G [10]. Taken together, these data demonstrate that the heart expresses a heterogeneous population of ankyrin-G isoforms that localize to the intercalated disc and/or Z-lines of individual myocytes.

### Identification of novel *Ank3* exons and splice variants in cardiac mRNA

**Fig 2:**
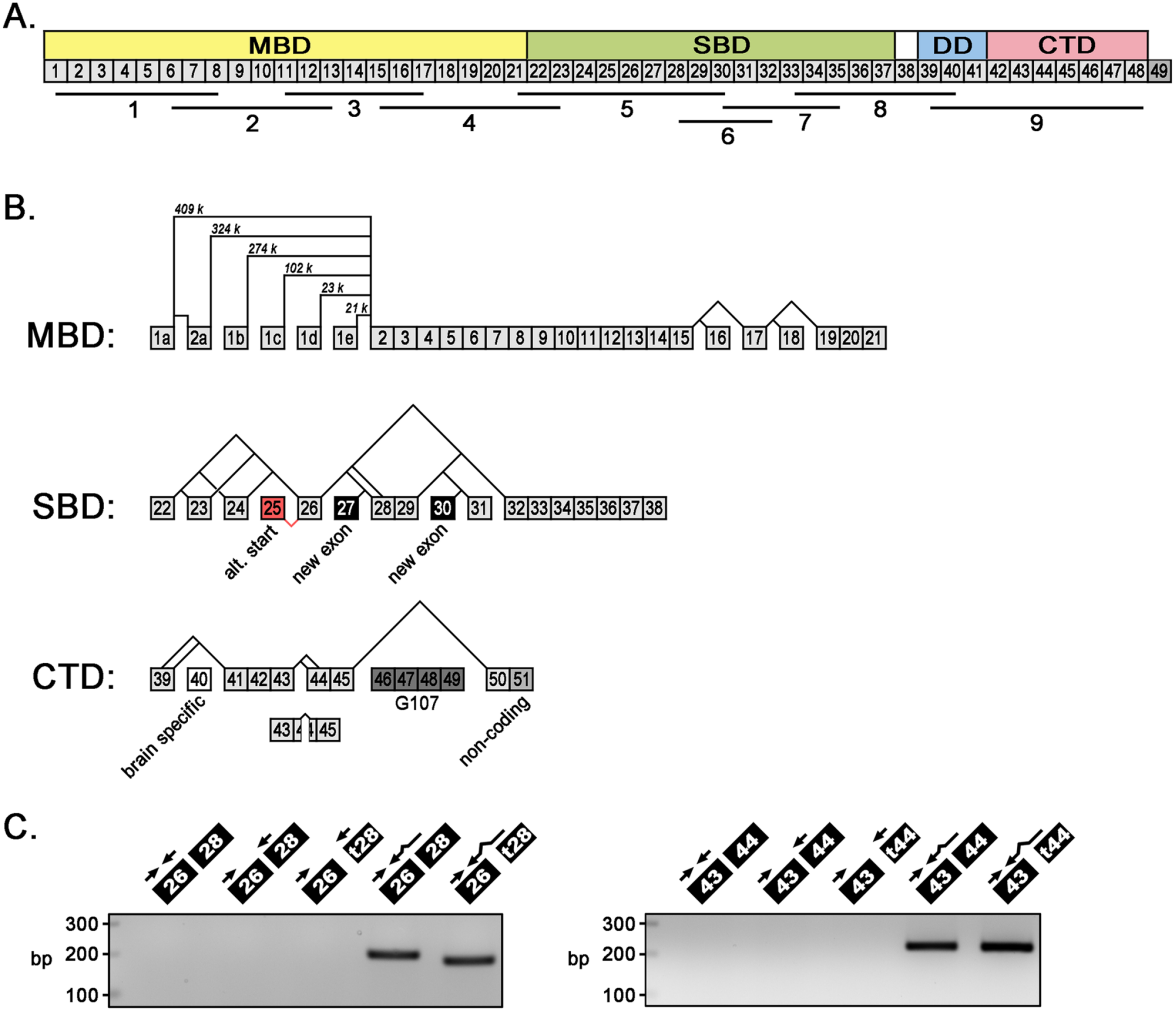
Exon organization and alternative splicing of *Ank3* gene. (**A**) Diagram of ankyrin-G exon organization and nine overlapping PCR primer sets used to amplify cardiac-specific *Ank3* alternative transcripts. Ankyrin functional domains are indicated. MBD: membrane-binding domain, SBD: spectrin-binding domain, DD: death domain, CTD: C-terminal domain. The large (7694 bp) brain-specific exon corresponds to exon 38. (**B**) Diagram of alternative *Ank3* splice variants encoding the MBD, SBD, and CTD. In the MBD, 5 alternative first exons (1a-1e) were detected. Lengths of intervening intronic sequences are labeled in kilobases (k). In the SBD, two novel exons (27 and 30) were identified and exon numbering has been adjusted accordingly (exon 40 is now the brain-specific exon). Exon 25 (in red) encodes the alternative start site for truncated ankyrin-G isoforms that lack MBDs (e.g. AnkG-107). In the CTD, alternative splicing of exons 46–49 (highlighted in gray) was not addressed in this study. (**C**) Full-length exon-exon boundary spanning primers are necessary to amplify PCR products: *Ank3* splice junctions of exons 26/28 and 26/t28 (truncated) (first panel) and exons 43/44 and 43/t44 (second panel).

To examine *Ank3* mRNA expression and identify novel alternative splice variants, we performed reverse-transcriptase PCR on ventricular mRNA using nine overlapping primer sets (Table 1) that spanned the length of the *Ank3* gene (Fig 2A). The integrity of the mRNA was evaluated by ethidium bromide staining of a 1% agarose gel. The presence of sharp 28S and 18S rRNA bands at a ratio of 2:1 and the absence of low molecular weight smears demonstrate that the mRNA was intact and not degraded (S1 Fig). We identified a variety of novel alternative *Ank3* mRNA transcripts including: 10 variants of the membrane-binding domain, 12 iterations of the spectrin-binding domain, and 6 permutations of the C-terminal regulatory domain (Fig 2B). The splicing patterns include exon-skipping (exon 16 in MBD), use of alternative 5’-donor site (exon 39 in CTD) or alternative 3’-acceptor site (exon 28 in SBD), and excision of internal exonic sequence (exon 44 in CTD). We also identified two new exons in the spectrin-binding domain (exons 27 and 30). Neither exon alters the open reading frame nor do they disrupt the minimal spectrin-binding domain. Table 2 includes an updated nomenclature for *Ank3* exon organization in addition to the lengths of individual exons and the intervening introns. Transcripts encoding the muscle-specific ankyrin-G isoforms that lack the membrane-binding domain (e.g. G107) initiate at the alternative start site in exon 25 and include exons 46–49. We have limited the scope of this study to the alternative splicing of full-length *Ank3* transcripts.

**Table 1:** *Ank3* primer sets.

#	5’ primer	3’ primer
1	ATGAGTGAAGAGCCAAAGGAGAAG	GCAACGTGTAAGGGAGTGATGTC
2	CTGCTCGAGAACGACACGAAGG	GAGCCCCATCTTGGACCAGATAC
3	GGTGCATCTATTCAAGCCGTAACC	GTTGACATTCGCGTTTCTACTCAG
4	GCCAAATACGGAAAGCTTGAAGTC	CTGAGATATATTCCCCATCACTGAGC
5	GAAGGTCGTGACGGAGGAAATTATG	CCGAAGGACGATAAGTTCTCTCTCC
6	CAGCGGACACGTTAGATAATGTGAAC	CTTGTACCCATTGGACACGCCTTC
7	CTTATCGTCCTTCGGAGCGAGAAC	CAAGCTGCTGTCCTCCTTTGG
8	GTGTGTCTTTCACAACCAACGTTTCAG	CCAGCAGAGTTACAATGTCTATCCGG
9	GATATCAGGATGGCGATAGTAG	GATTTCCTTCTTCGTCTTCACC

**Table 2:** *Ank3* exon organization.

Exon #	Previous exon #	5’-splice	3’-splice	Length /CDS (bp)	3’ intron (bp)	Domain	Comments
1a			AGCAG/gt	357/57	85099	MBD	Start
2a		ag/GGTGA	AAAAG/gt	39	49954	MBD	
1b	1		AAAAG/gt	577/63	172527	MBD	Start
1c			AAAAG/gt	861/114	79015	MBD	Start
1d			AAGAA/gt	358/36	1942	MBD	Start
1e			AGAGG/gt	343/24	20966	MBD	Start
2b	2	ag/TCTGA	ACCAG/gt	102	430	MBD	ANK Repeat 1
3	3	ag/AATGG	CAAAG/gt	99	199	MBD	ANK Repeat 2
4	4	ag/AAAGG	CTCAG/gt	99	6425	MBD	ANK Repeat 3
5	5	ag/AATGG	CAGAG/gt	99	6501	MBD	ANK Repeat 4
6	6	ag/GACGG	CAAAG/gt	186	1924	MBD	ANK Repeat 5, 6
7	7	ag/AGTGG	CACGG/gt	99	25689	MBD	ANK Repeat 7
8	8	ag/AATGA	CCAGG/gt	99	17180	MBD	ANK Repeat 8
9	9	ag/GACGG	CCAAG/gt	99	3518	MBD	ANK Repeat 9
10	10	ag/AATGG	CCCTG/gt	198	3590	MBD	ANK Repeat 10, 11
11	11	ag/AATGG	CCGAG/gt	99	2941	MBD	ANK Repeat 12
12	12	ag/TCGGG	ATGTG/gt	99	1964	MBD	ANK Repeat 13
13	13	ag/AGAGG	CTAAG/gt	99	2369	MBD	ANK Repeat 14
14	14	ag/GATGA	CAAAG/gt	198	4376	MBD	ANK Repeat 15, 16
15	15	ag/AAGGG	GGAAG/gt	99	293	MBD	ANK Repeat 17
16	16	ag/AGCGG	CAAAG/gt	99	7084	MBD	ANK Repeat 18
17	17	ag/AATGG	ATAAG/gt	198	925	MBD	ANK Repeat 19, 20
18	18	ag/AGCGG	CAAAG/gt	99	4497	MBD	ANK Repeat 21
19	19	ag/ATGGG	CGAAG/gt	99	89	MBD	ANK Repeat 22
20	20	ag/AATGG	CTGTG/gt	99	667	MBD	ANK Repeat 23
21	21	ag/AATGG	CCACT/gt	96	5108	MBD	ANK Repeat 24
22	22	ag/ACCAT	TGAAG/gt	73	177	SBD	
23	23	ag/TAAGG	AGAAG/gt	63	15927	SBD	
24	24	ag/GTGAT	GAAAA/gt	54	1628	SBD	
25	25		ACACA/gt	384/16	1503		3’ intron to ATG of E25/alt. start
26	26	ag/GTGAA	GCCAG/gt	124	4575/4590	SBD	
27		ag/TCCCA	ATAAG/gt	12	2935	SBD	new exon
28	27	ag/CCTCC	AGCAG/gt	103	18455	SBD	
28’		ag/TTCGG	AGCAG/gt	88	18455		5’-truncated exon 28
29	28	ag/CACCT	TCTGG/gt	107	2381	SBD	
30		ag/CTGCT	TCAAG/gt	33	2453	SBD	new exon
31	29	ag/GTTTC	TTAGG/gt	225	2767	SBD	ZU5 motif
32	30	ag/CCCCG	TGAAG/gt	155	14649	SBD	ZU5 motif
33	31	ag/AACTC	TCCAG/gt	212	2114	SBD	
34	32	ag/GCTCA	CACAG/gt	208	1457	SBD	
35	33	ag/GAGGC	GCCAG/gt	97	661	SBD	
36	34	ag/ATTCT	TTGAG/gt	229	618	SBD	
37	35	ag/GTTCT	TCAAG/gt	126	828	DD	
38	36	ag/ATCAG	AAAAG/gt	123	1721	DD	
39	37	ag/GCTGA	CATGA/gt	82	3891	DD	
39’		ag/GCTGA	TTTAC/gt	46	3927		3’-truncated exon 39
40		ag/TTGAA	GACAG/gt	7694	589		brain-specific exon
41	38	ag/GTCCG	GACAG/gt	75	3648	CTD	
42	39	ag/AGCTG	CACAA/gt	132	1128	CTD	
43	40	ag/CTGAT	TGATG/gt	144	2424/3012	CTD	
44	41	ag/GTCAC	GACAG/gt	679	2008	CTD	
44’		ag/GTTGG	GACAG/gt	91	2008	CTD	5’-truncated exon 44
45	42	ag/CTCTG	CTAAG/gt	382	1808	CTD	
46		ag/CAGGT	ACAAG/gt	54	1924	CTD	18-aa insert
47		ag/GACTT	TAAAG/gt	87	5107	CTD	76-aa insert
48		ag/GGGGA	GAAAA/gt	84	135	CTD	76-aa insert
49		ag/ATCAC	ACGTG/gt	57	1321	CTD	76-aa insert
50	43	ag/CAGGG	TCCAG/gt	86	8178	CTD	Stop
51	44	ag/GACCA	TTCCT	3605			Non-coding

Table includes current and previous exon numbers (NM_146005.3), adjacent nucleotide sequences to 5’- and 3’- splice junctions, exon length (and length of coding sequence CDS for first exons), intron length, functional domain encoded by the exon, and any additional comments. NCBI accession numbers for the first exons are as follows: exon 1a: BB614215, exon 1b: BX520427, exon 1c: CD802881, exon 1d: DV047480, exon 1e: AI614790, and exon 25: BB628284.

To determine the relative mRNA expression of particular *Ank3* transcripts, we designed exon-exon boundary spanning primers that selectively PCR amplify transcripts based on their unique exon junctions. Specifically, one PCR primer spans the junction of two adjacent exons and the full-length primer is required to amplify a PCR product (Fig 2C). PCR conditions (primer lengths, annealing temperatures) are optimized for quantitative real-time PCR analysis such that the efficiency of each primer set falls within the range of 90–110%. Nucleotide sequences, annealing temperatures, and primer efficiencies for all 28 primer sets are listed in S1 Table. We have previously used this technique to demonstrate the expression of alternative *ANK2* transcripts in mouse and human hearts [13].

### Expression of five *Ank3* alternative first exons in various tissues

**Fig 3:**
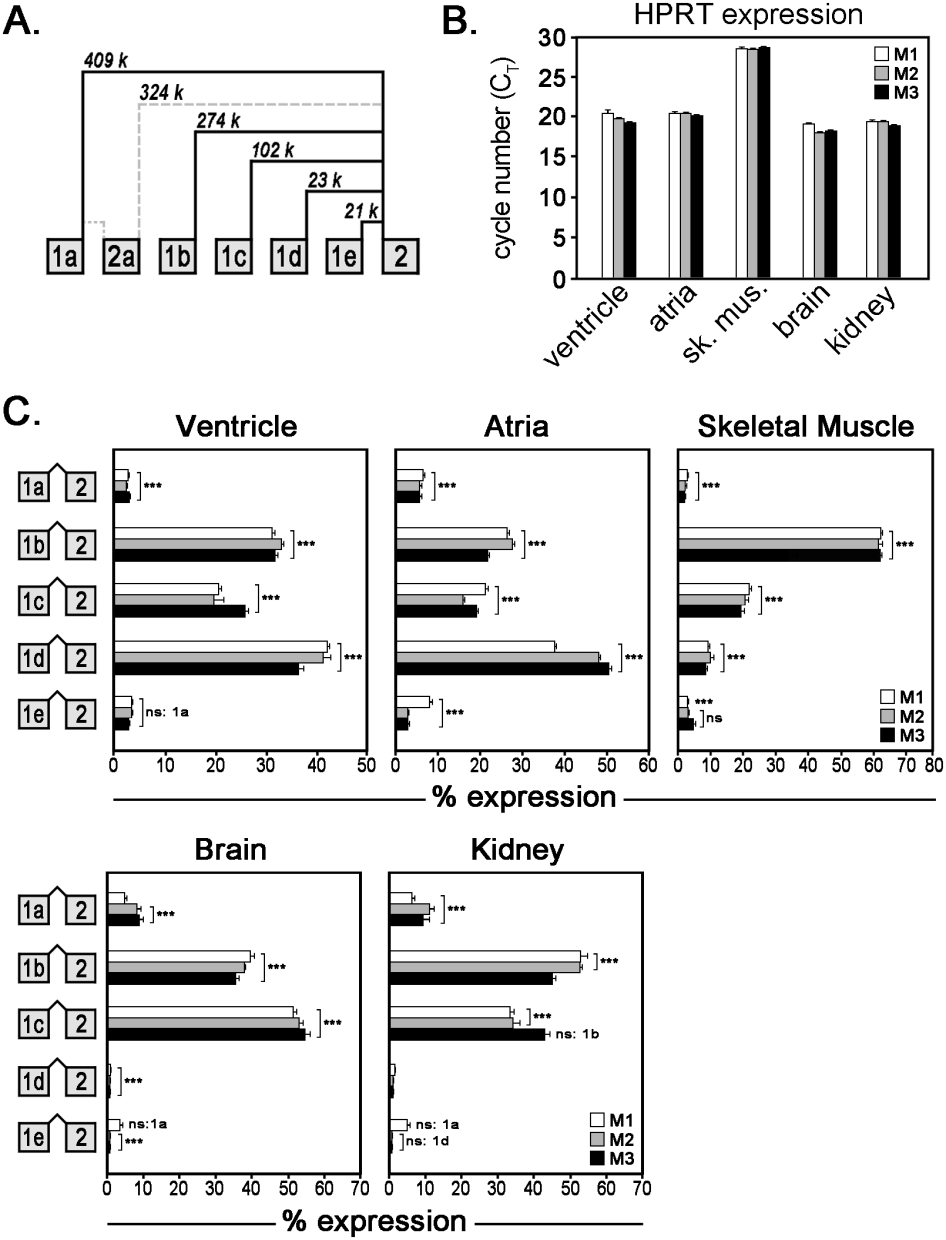
Relative mRNA expression of alternative *Ank3* first exons. (**A**) Diagram of alternative splicing of 5 first exons of the *Ank3* gene. Gray dashed lines represent rare alternative spliced junctions (<1%). (**B**) qt-PCR analysis of hypoxanthine-guanine phosphoribosyltransferase (HPRT) expression (measured as cycle threshold number) in ventricle, atria, skeletal muscle, brain, and kidney from three mice (labeled M1–M3). (**C**) Relative mRNA expression of alternative first exons was measured in heart (ventricle and atria), skeletal muscle, brain, and kidney by qt-PCR analysis. Bar graphs represent technical replicates of qt-PCR samples and error bars represent standard deviations. Statistical analysis was performed with one-way ANOVA with Tukey’s multiple comparison test (*** *p*-value ≤0.001, ns: not significant) to assess the significance of expression differences between different splice variants (i.e. 1a vs. 1b).

The *Ank3* gene has five first exons within ~409 kb of the second exon. To determine the relative expression of each first exon in heart, we performed quantitative real-time PCR analysis using exon-exon boundary spanning primers that selectively detect specific exon junctions. The expression of each first exon is represented relative to the expression of the other first exons. Therefore, the sum of expression of exons 1a, 1b, 1c, 1d, and 1e equals 100%. Rare alternative splice junctions (< 1%) are indicated by gray, dashed lines (Fig 3A) and not included in the summation, although the primary data is provided in S2 Table.

In heart, we compared the relative expression of the first exons in ventricular and atrial mRNA isolated from three mouse hearts. We found that *Ank3* transcripts with first exons 1b, 1c, or 1d are more abundant than transcripts with first exons 1a or 1e (Fig 3C). In addition to heart, *Ank3* gene products have been detected in numerous tissues including skin, skeletal muscle, lung, kidney, and brain [1–6, 20–22]. To determine if first exons display tissue-specific expression, we measured the relative expression of the first exons in mRNA transcripts isolated from kidney, brain, and skeletal muscle (whole tissue). In skeletal muscle, the relative expression of *Ank3* transcripts with first exon 1b is ~60%, 1c is ~20%, and 1d is ~10% (Fig 3C). Ank3 transcripts with first exons 1a and 1e are expressed less than 5% in skeletal muscle. In kidney and brain, we found that *Ank3* transcripts with first exons 1b and 1c are more abundant than transcripts with first exons 1a and 1e (Fig 3C). Interestingly, *Ank3* transcripts with first exon 1d were virtually undetectable in kidney and brain. Exon 1d was also undetected in mRNAs isolated from cerebellum and lung (data not shown). To validate the similarity of mRNA quantity and quality isolated from the different mouse tissues, we demonstrated equivalent expression of the housekeeping gene hypoxanthine-guanine phosphoribosyltransferase (HPRT) in mRNA samples from the ventricle, atria, skeletal muscle, brain, and kidney (Fig 3B).

### Cardiac expression of *Ank3* splice variants encoding ankyrin-G MBD

**Fig 4:**
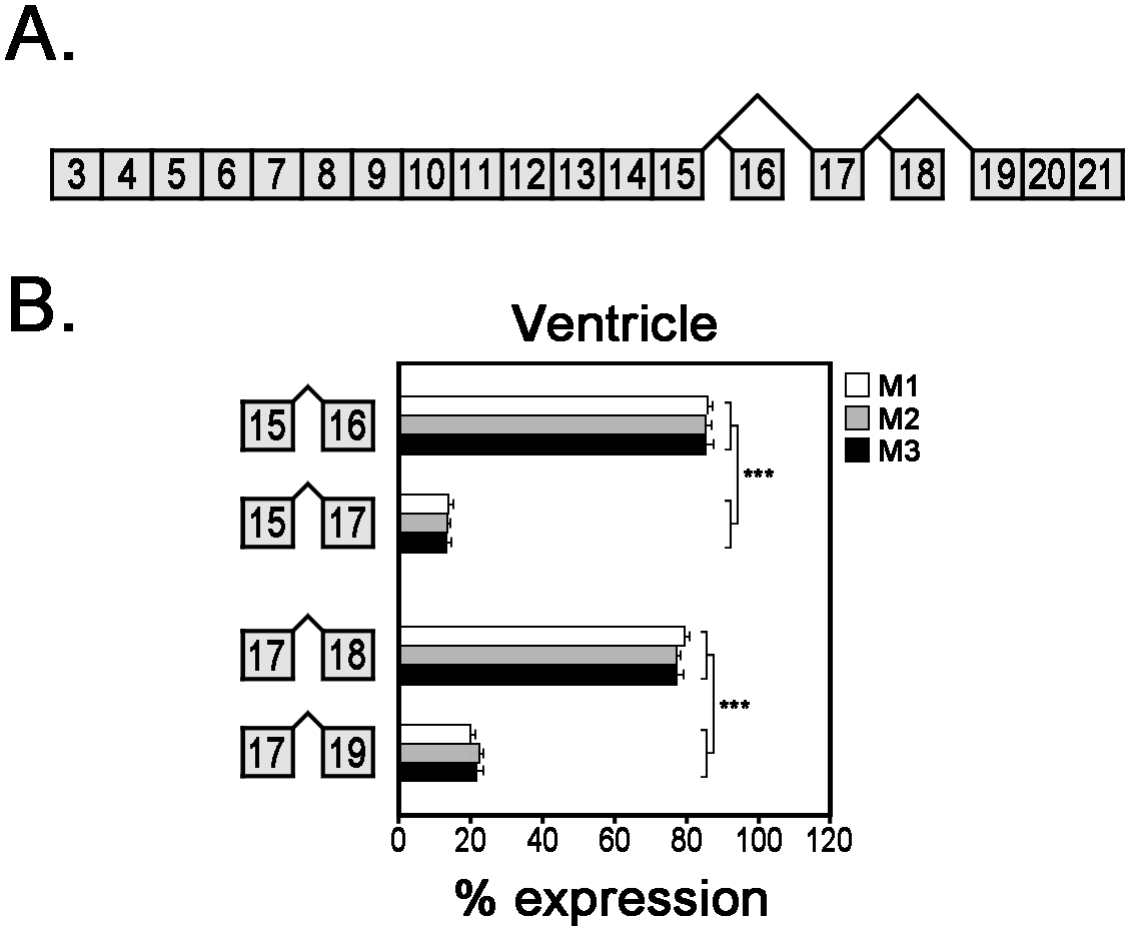
Relative mRNA expression of alternative *Ank3* transcripts encoding ankyrin-G MBD. (**A**) Diagram of *Ank3* alternative splicing in ankyrin-G MBD. (**B**) Expression of *Ank3* mRNA transcripts ± exon 16 or ± exon 18 was measured in three mouse hearts using qt-PCR analysis with exon-exon boundary spanning primers. Bar graphs represent technical replicates of qt-PCR samples and error bars represent standard deviations. Statistical analysis was performed with unpaired Student’s T-test (*** *p*-value ≤0.001).

*Ank3* exons 1–21 encode the ankyrin-G MBD (Table 2). During the screen for alternative transcripts, we identified four alternative splice variants (± exon 16 and ± exon 18) of the MBD (Fig 4A). To measure the relative expression of these alternative splice variants, we performed quantitative real-time PCR using exon-exon boundary spanning primers on mRNAs isolated from mouse ventricular tissue. We found that splice variants including exon 16 or exon 18 are about four-fold more abundant than transcripts that lack these exons (Fig 4B).

### Cardiac expression of *Ank3* splice variants encoding ankyrin-G SBD

**Fig 5:**
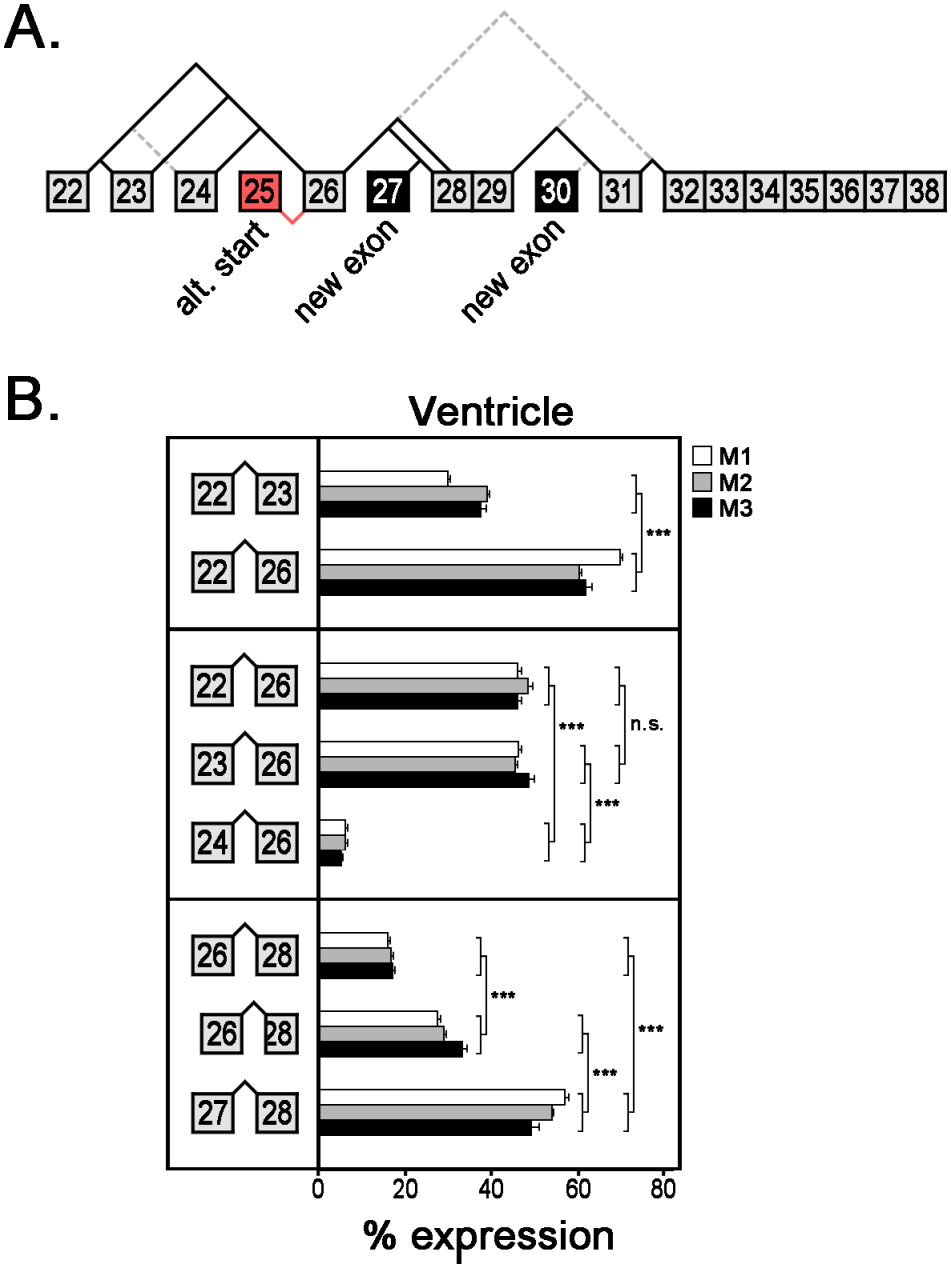
Relative mRNA expression of alternative *Ank3* transcripts encoding ankyrin-G SBD. (**A**) Diagram of *Ank3* alternative splicing in ankyrin-G SBD. Newly identified exons are labeled in black and the alternative first exon (E25) is labeled in red. Gray dashed lines represent rare alternative spliced junctions (<1%). (**B**) Expression of various *Ank3* mRNA transcripts was measured in three mouse hearts using qt-PCR analysis with exon-exon boundary spanning primers. Bar graphs represent technical replicates of qt-PCR samples and error bars represent standard deviations. Statistical analysis was performed using one-way ANOVA with Tukey’s multiple comparison test or unpaired Student’s T-test (*** *p*-value ≤0.001, ns: not significant).

During the screen for cardiac-specific *Ank3* transcripts, we identified two new exons (27 and 30) in the SBD (Fig 5A and Table 2). We also identified 12 novel alternative spliced transcripts. We measured the relative expression of each splice junction by quantitative real-time PCR. Four splice junctions (E22/24, E26/32, E30/31, E29/32) are so rare (< 1%) that we excluded them from the determination of relative mRNA expression, although the primary data is provided S2 Table. We identified three *Ank3* transcripts with unique 3’-splice junctions with exon 22: E22/23, E22/24, and E22/26. Expression of E22/24 is less than 1%, while E22/26 is ~1.5 fold more abundant than E22/23 (Fig 5B). We also identified three *Ank3* transcripts with unique 5’-splice junctions with exon 26: E22/26, E23/26, and E24/26. While both E22/26 and E23/26 are equally abundant (~45% each), E24/26 is much less abundant (~5%). Lastly, we identified three *Ank3* transcripts with unique 5’-splice junctions with exon 28: E26/28, E26/tr28 (tr:truncated), and E27/28. Interestingly, exon 27 is one of the newly identified exons and transcripts containing this exon (E27/28) are the most abundant (~55–60%), followed by transcripts with the splice junctions of E26/tr28 (~25%) and then E26/28 (~15%).

### Two ankyrin-G SBD isoforms lack β-spectrin binding

**Fig 6:**
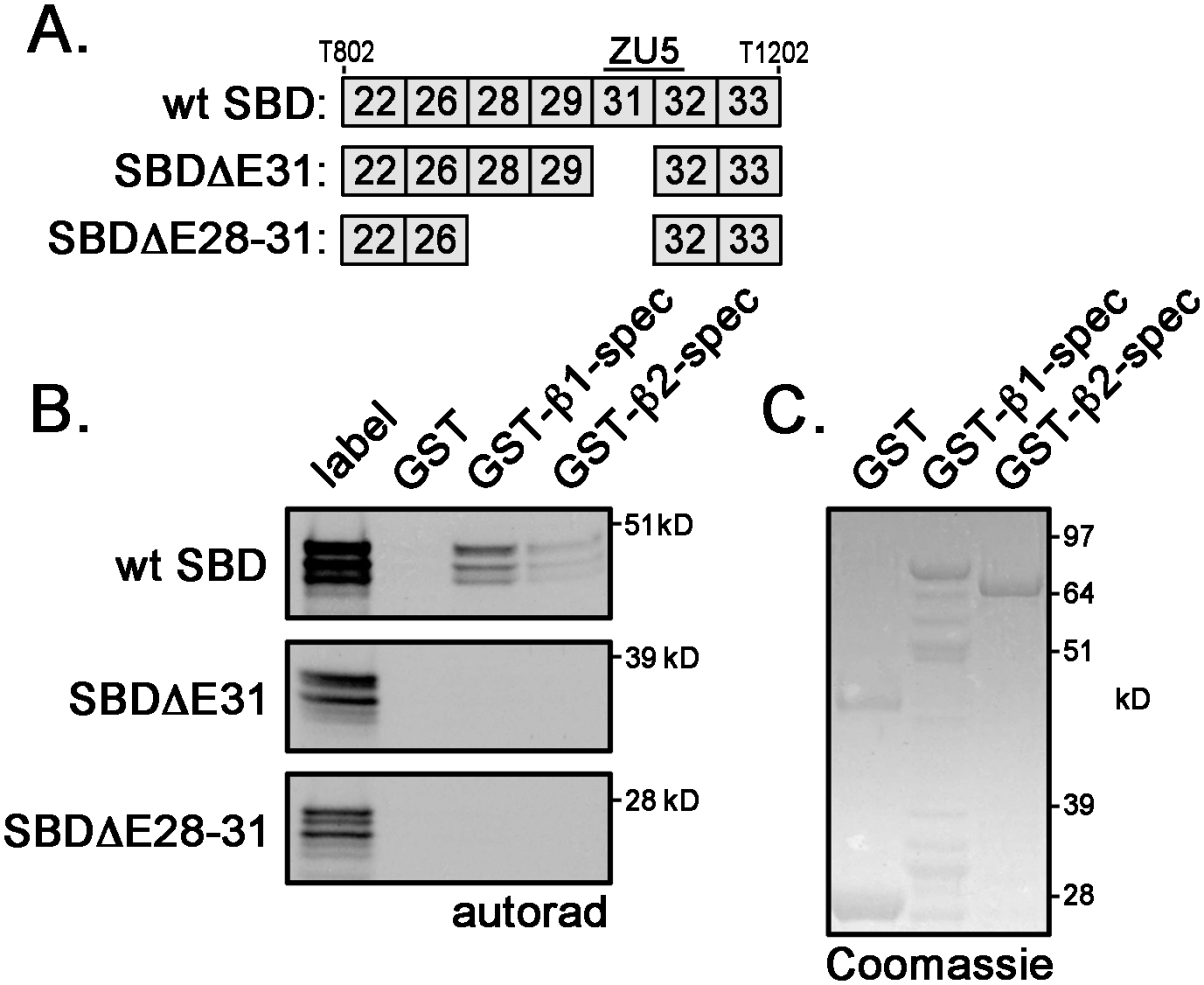
Alternative ankyrin-G SBD isoforms do not bind β-spectrin. (**A**) Diagram of *Ank3* exon organizations for two rare ankyrin-G SBD variants (ΔE31 and ΔE28–31). Both variants lack a portion of the ZU5 motif. (**B**) β1-spectrin and β2-spectrin (repeats 13 to 17) binding to ankyrin-G SBDs (wildtype, Δ31, Δ28–31) was measured by *in vitro* binding assays. Label is the unbound *in vitro* translated SBD products. (**C**) Coomassie Blue Stain demonstrates equal loading of GST-fusion proteins β1- and β2-spectrins.

In the spectrin-binding domain, the ZU5 motif represents the minimal β-spectrin binding domain and is encoded by exons 31 and 32 [23]. We identified two novel alternative *Ank3* splice variants that lacked exon 31. While these transcripts in heart are rare (< 1%) (S2 Table), we evaluated β-spectrin binding to GST-fusion proteins of these SBD isoforms (SBDΔE31 and SBDΔE28–31) (Fig 6A). The ankyrin-binding domain has been mapped to spectrin repeats 14 and 15 [24–26]; therefore, we generated GST-fusion proteins of spectrin repeats 13 through 17 for both β1- and β2-spectrins. Briefly, *in vitro* translated and ^35^S-labelled SBD protein fragments were incubated with GST, GST β1-spectrin, or GST β2-spectrin. Glutathione sepharose precipitated radiolabelled protein complexes that were resolved by SDS-PAGE and visualized by autoradiography. While β1- and β2-spectrins bind wild-type SBD, the SBD displays greater binding capacity for β1-spectrin than for β2-spectrin (Fig 6B). In contrast, neither SBDΔE31 nor SBDΔE28–31 bound to spectrin demonstrating that partial loss of the ZU5 motif completely disrupts SBD binding to β-spectrin. Coomassie Blue Stain demonstrated equal loading of GST fusion proteins β1- and β2-spectrin (Fig 6C).

### Cardiac expression of *Ank3* splice variants encoding ankyrin-G CTD

The focus of this study is to identify novel variants of full-length ankyrin-G. *Ank3* exons 46, 47, 48, and 49 encode an obscurin-binding domain that is uniquely expressed in truncated ankyrin-G isoforms that were initially characterized in skeletal muscle (e.g. G107) [2–4]. We were unable to detect these exons in full-length *Ank3* transcripts in heart; therefore, the characterization of alternative splicing of this domain was excluded from this study.

**Fig 7:**
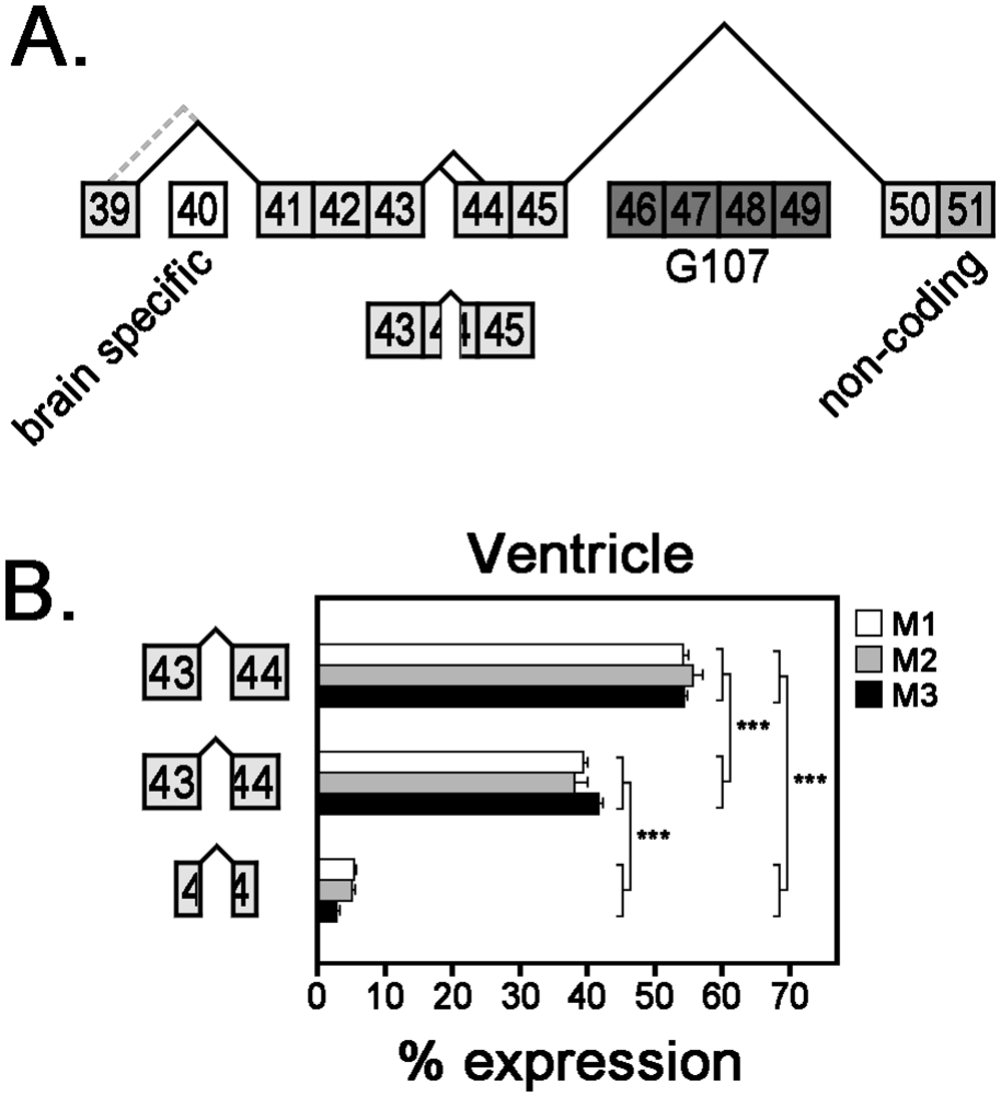
Relative mRNA expression of alternative *Ank3* transcripts encoding ankyrin-G CTD. (**A**) Diagram of *Ank3* alternative splicing in ankyrin-G CTD. Exon 40 is the large (7694 bp) brain-specific exon, while exon 51 contains non-coding sequence. Exons 46–49 are expressed in truncated ankyrin-G isoforms that lack membrane-binding domains. Gray dashed lines represent rare alternative spliced junctions (<1%). (**B**) Expression of various *Ank3* mRNA transcripts with different iterations of exon 44 (full-length, 5’-truncated, medially truncated) was measured in three mouse hearts using qt-PCR analysis with exon-exon boundary spanning primers. Bar graphs represent technical replicates of qt-PCR samples and error bars represent standard deviations. Statistical analysis was performed with one-way ANOVA with Tukey’s multiple comparison test (*** *p*-value ≤0.001).

We identified six novel *Ank3* splice variants of the CTD (Fig 7A). Exon 40 was never detected in any cardiac transcripts and we identified two splice junctions that excise this brain-specific exon. The splice junction of E39/41 is predominant in heart and the use of an alternative 5’-donor site within exon 39 is exceedingly rare (< 1%) (S2 Table). We identified three alternative splice variants of exon 44: E43/44, E43/tr44, and a medial excision of 267 base pairs from exon 44. Exon 44 is 679 bp in length and the 5’-truncation removes 588 bp (tr44). The splice variant E43/44 is more abundant than E43/tr44 (~55% versus ~40%), while the splice variant that lacks 267 base pairs in the middle of exon 44 is less common (~5%) (Fig 7B).

### Exogenous ankyrin-G CTD isoforms display similar subcellular expression as endogenous ankyrin-G

**Fig 8:**
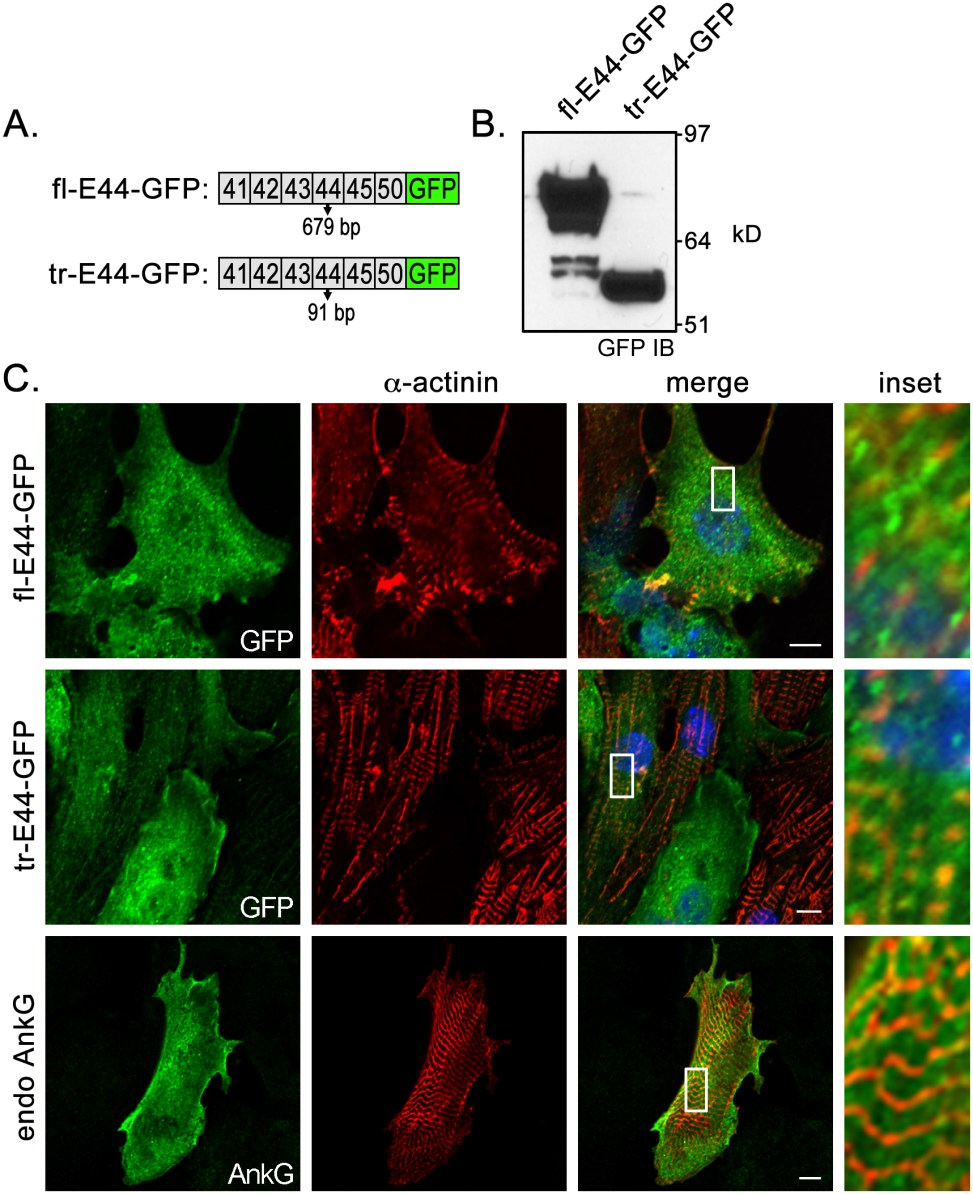
Subcellular localization of ankyrin-G CTD isoforms in neonatal cardiomyocytes. (**A**) Diagram of lenti-viral GFP-tagged CTD variants with full-length or truncated exon 44. (**B**) GFP immunoblot analysis demonstrating expression of lenti-viral constructs. (**C**) Immunofluorescent co-localization of GFP-tagged CTD variants (fl-E44-GFP and tr-E44-GFP) with Z-line resident protein α-actinin in neonatal cardiomyocytes. Bottom panels represent localization of endogenous ankyrin-G in reference to α-actinin. Scale bar represents 10 microns.

Ankyrin-G is localized to the intercalated disc and Z-lines in adult ventricular cardiomyocytes (Fig 1B–1C). We have previously demonstrated that the C-terminal domain of ankyrin-B plays an important role in targeting this related adaptor protein to the sarcomeric M-line in cardiomyocytes [27]. To assess whether the C-terminal domain of ankyrin-G contains targeting motifs, we evaluated the subcellular localization of the two most abundant CTD variants in virally transduced neonatal cardiomyocytes. Lenti-viral expression in neonatal cardiomyocytes is preferable to adult cardiomyocytes because they survive in culture for 7–10 days compared to adult myocytes, which survive for ~24 hours. Lenti-viral constructs with GFP tags were made of the ankyrin-G CTD containing the full-length exon 44 (679 bps) or the 5’-truncated exon 44 (lacking 588 bps) (Fig 8A). Expression of lenti-viral constructs was confirmed by immunoblot analysis of the GFP tag (Fig 8B). We evaluated the co-localization of these CTD variants to α-actinin in neonatal cardiomyocytes. Both variants displayed diffuse cytosolic localization and lacked the defined patterning of sarcomeres. Moreover, both variants displayed similar subcellular distributions as endogenous ankyrin-G, which was detected using the polyclonal ankyrin-G antibody (Fig 8C). One of the limitations of neonatal cardiomyocytes as a model system is that they lack fully developed subcellular domains, which may be required for the proper targeting of ankyrin-G.

## Discussion

This study is the first to describe a comprehensive analysis of *Ank3* expression in heart. We identified two new exons and 28 novel alternative splicing events in the *Ank3* gene. An update to the *Ank3* exon organization and nomenclature is provided in Table 2. Using quantitative real-time PCR with exon-exon spanning primers, we demonstrate that alternative *Ank3* splice variants are expressed at similar levels in three separate hearts. Moreover, we find that expression of *Ank3* transcripts initiated with exon 1d is restricted to the heart and skeletal muscle as these transcripts are undetectable in brain, kidney, cerebellum, and lung.

The majority of alternative splicing is situated within the coding region of the spectrin-binding domain, specifically within the exons immediately 5’ of the minimal spectrin-binding domain ZU5A, which is encoded by exons 31 and 32 [28, 29] (Figs 2 and 5). The newly identified exons 27 and 30 are also alternatively spliced, but neither exon alters the open-reading frame of ankyrin-G and exon 30 is rarely expressed in cardiac transcripts (Fig 5 and Table 2). Interestingly, exon 30 is included in three expressed sequence tags (CO430616, CF745086, CV555908) that were isolated from neural tissue. We identified two rare alternative splice variants that remove exon 31 (junctions of exons 26 to 32 and exons 29 to 32). *In vitro* translated products of these SBD isoforms lacked binding to both β1- and β2-spectrins (Fig 6). Moreover, wild-type ankyrin-G SBD bound more β1-spectrin than β2-spectrin. Ankyrin-G SBD binding to β-spectrin isoforms (β1, β2, β3, and β4) has yet to be studied extensively, much less the influence of alternative splicing on this process.

We demonstrate that the heart expresses five distinct ankyrin-G isoforms by immunoblot analysis. Imaging of individual cardiomyocytes reveals the expression of two ankyrin-G subpopulations. One population co-localizes with the voltage-gated sodium channel Na_V_1.5 at the intercalated disc (ICD), while the other population localizes to the Z-line. Interestingly, the ICD population is detected using the ankyrin-G monoclonal antibody, while the Z-line population is detected with the ankyrin-G polyclonal antibody. These differences may be the result of different antigens used for antibody production. The monoclonal antibody was generated against an antigen encoded by exons 26, 28, 29, 31–39, 41–45, and 50. In addition, splicing variations present in the antigen include 5’-truncation of exon 28, 3’-truncation of exon 39, and medial excision of 267 base pairs from exon 44. The antigen for the polyclonal antibody consisted of the death and C-terminal domains, which included the full-length exon 44.

The expression of numerous ankyrin-G isoforms in a specific tissue is quite common. Understanding the function of each isoform remains a challenge, but insights into an isoform’s function may be inferred from its subcellular localization. In kidney and muscle, the truncated AnkG119 isoform is located in the Golgi apparatus and is necessary for transport of sodium potassium ATPase (NKA) to the endoplasmic reticulum [1, 30]. Many alternative ankyrin-G isoforms that lack membrane-binding domains are expressed in skeletal muscle [2–4]. The obscurin-binding domains encoded by *Ank3* exons 46–49 in the C-terminal domain regulate the sarcolemmal localization of these isoforms [2]. While a previous study demonstrated skeletal muscle expression of a full-length ankyrin-G isoform with exons 46–49 [3], we were unable to detect a similar isoform in cardiac muscle.

To evaluate whether *Ank3* exon 44 encoded any targeting motifs, we examined the subcellular localization of the two most abundant CTD variants in neonatal cardiomyocytes. Neither isoform displayed sarcomeric localization, similar to expression of endogenous ankyrin-G (detected with the polyclonal antibody) (Fig 8C). While neonatal cardiomyocytes are viable to express proteins introduced by lenti-virus, they lack the elaborate T-tubular network found in adult cardiomyocytes. Proper subcellular targeting of ankyrin-G most likely requires fully developed membrane domains in adult myocytes.

Given the diversity of functions and subcellular localizations ascribed to ankyrin-G, it is not surprising that the heart expresses multiple ankyrin-G isoforms. In ventricular cardiomyocytes, ankyrin-G expression overlaps the expression of voltage-gated sodium channels (Na_V_) at the intercalated disc and T-tubules. Interestingly, the voltage-gated sodium channels display differential subcellular localization with Na_V_1.5 predominantly expressed at the intercalated disc, while the other sodium channels (Na_V_1.1, 1.3, and 1.6) are predominantly localized to the T-tubules [31–33]. In addition to interacting with Na_V_1.5 at the intercalated disc, ankyrin-G has also been shown to interact with plakoglobin and connexin 43 [11]. Whether the intercalated disc expresses a heterogeneous population of ankyrin-G isoforms is not known, but such a scenario could account for the diversity of ankyrin-G interactions. Finally, while it has been shown that ankyrin-G is required for the retention of dystrophin and β-dystroglycan at the costameres in skeletal muscle (and presumably cardiac muscle) [7, 8], the cDNA of this particular ankyrin-G isoform has yet to be characterized. Future research efforts will focus on identifying full-length *Ank3* transcripts in heart and evaluating these isoforms for specific functions.

In closing, there are tremendous opportunities for discovery in examining the splicing regulation of ankyrin genes. Our data suggests that the *Ank3* gene is subject to complex transcriptional regulation in the heart. We propose that alternative splicing of the *Ank3* gene results in a diverse population of ankyrin-G isoforms with each isoform aggregating a unique set of proteins that impart specific functionality to unique cardiomyocyte domains such as the intercalated disc, T-tubule, or costamere.

## Supporting Information

S1 Fig EtBr-stained agarose gel of mRNA isolated from 3 mouse hearts. (TIF)

S1 Table Primer sequences for qt-PCR analysis of *Ank3* splice junctions. (DOCX)

S2 Table Average C_T_ values of rare *Ank3* transcripts in 3 mouse hearts. (DOCX)
