# Supplementary figures and images for: Exon Organization and Novel Alternative Splicing of Ank3 in Mouse Heart
Source: PLoS One. 2015 May 29;10(5):e0128177. doi: 10.1371/journal.pone.0128177 (PMC4449188; doi:10.1371/journal.pone.0128177)

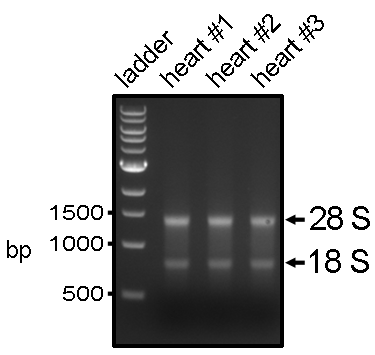

Supplement: S1 Fig — (TIF) [file pone.0128177.s001.tif]
