# Supplementary material for: Exon Organization and Novel Alternative Splicing of Ank3 in Mouse Heart
Source: PLoS One. 2015 May 29;10(5):e0128177. doi: 10.1371/journal.pone.0128177 (PMC4449188; doi:10.1371/journal.pone.0128177)
Supplement: S1 Table — (DOCX) [file pone.0128177.s002.docx]

**S1 Table. Primer sequences for qt-PCR analysis of *Ank3* splice junctions.**

| Primer Set | 5' primer | 3' primer | Size (bp) | Tm (°C) | Eff. |
| --- | --- | --- | --- | --- | --- |
| E1a/E2a | GATCTTGAGCAG/ggtgactttgg | gtggacgtcaacatctgtaaccag | 153 | 64 | 99.66 |
| E1a/E2b | GATCTTGAGCAG/tctgatgccaac | gtggacgtcaacatctgtaaccag | 114 | 62 | 102.43 |
| E1b/E2b | GAGGAAAGGAAAAAAG/tctgatgccaac | gtggacgtcaacatctgtaaccag | 118 | 66 | 95.30 |
| E1c/E2b | ATCGCAAGAAAAAG/tctgatgccaac | gtggacgtcaacatctgtaaccag | 116 | 64 | 100.01 |
| E1d/E2b | CTTCCCAGAAGAA/tctgatgccaac | gtggacgtcaacatctgtaaccag | 115 | 66 | 92.53 |
| E1e/E2b | CTTGCTAAGAGG/tctgatgccaac | gtggacgtcaacatctgtaaccag | 114 | 66 | 95.68 |
| E15/E17 | CCGCAGGGAAG/aatggctatac | gtggacgtcaacatctgtaaccag | 209 | 66 | 105.58 |
| E16/E17 | CAGCCGCAAAG/aatggctataca | gtggacgtcaacatctgtaaccag | 209 | 64 | 97.43 |
| E17/E18 | aatggctatacaccactgcacatc | CTGAGCAATAAG/atgggctac | 209 | 66 | 110.68 |
| E17/E19 | aatggctatacaccactgcacatc | CTGAGCAATAAG/agcggtctcac | 206 | 56 | 91.29 |
| E22/E23 | gaacacagctctggccatcg | TCAGACGATGAAG/taaggaaagccag | 177 | 66 | 109.59 |
| E22/E24 | CAGACGATGAAG/gtgataaatgca | gttacgtaggcttcagtcttgga | 176 | 60 | 96.65 |
| E22/E26 | ggttacatctcggtggttgac | GTTTTGGTGAAAA/gtgaagatgccat | 143 | 66 | 99.66 |
| E23/E26 | ggttacatctcggtggttgac | GGTGAAGAAG/gtgaagatgccat | 206 | 64 | 105.12 |
| E24/E26 | ggttacatctcggtggttgac | GACGATGAAG/gtgaagatgccat | 263 | 62 | 105.12 |
| E26/E27 | gtgaagatgccatcacaggggac | CTTATCTTGGGA/ctggcagaacgg | 136 | 68 | 96.93 |
| E26/E28 | TTCTGCCAG/cctccgctcctt | gcagcggacacgttagataatgtg | 190 | 64 | 104.44 |
| E26/trE28 | TTCTGCCAG/ttcggataggtcc | gcagcggacacgttagataatgtg | 175 | 64 | 90.94 |
| E26/E32 | GTTCTGCCAG/ccccgtcatt | catcagtttgacagtaaaaacgaagacctc | 137 | 57 | 96.26 |
| E29/E31 | gataggtcctacaccttgaacag | AGTATGACTCAAG/gtttctggttagc | 203 | 60 | 95.30 |
| E30/E31 | gataggtcctacaccttgaacag | GTGCATTCTGG/gtttctggttag | 237 | 60 | 113.01 |
| E29/E32 | TGCATTCTGG/ccccgtcatt | gcttctcaatggcatggatgaag | 165 | 61 | 91.64 |
| E31/E32 | ACAATTTTTAGG/ccccgtcattgt | gcttctcaatggcatggatgaag | 167 | 64 | 102.21 |
| E39/E41 | GAACCCAGCATGA/gtccgcag | gaccagagacggaaagaatgc | 215 | 64 | 90.08 |
| E39tr/E41 | CTAGCTTTAC/gtccgcag | gaccagagacggaaagaatgc | 212 | 68 | 94.17 |
| E43/E44 | caaccaaatacgtgtggaaaatcc | CAGTTGATG/gttggcagaacg | 251 | 68 | 103.31 |
| E43/trE44 | caaccaaatacgtgtggaaaatcc | CAGTTGATG/gtcacccttcct | 251 | 64 | 106.05 |
| ½-E44 | gtgatatctctagcatagagtctcc | TGTCAGAGCGA/gttggcagaac | 237 | 60 | 113.01 |

Table includes amplicon length, annealing temperature (Tm), and primer efficiencies. The splice junction is indicated by the transition from all capital letters to lower case letters.
