# Supplementary material for: Exon Organization and Novel Alternative Splicing of Ank3 in Mouse Heart
Source: PLoS One. 2015 May 29;10(5):e0128177. doi: 10.1371/journal.pone.0128177 (PMC4449188; doi:10.1371/journal.pone.0128177)
Supplement: S2 Table — (DOCX) [file pone.0128177.s003.docx]

**S2 Table. Average C_T_ values of rare *Ank3* transcripts in 3 mouse hearts.**

|  | Mouse 1 | | Mouse 2 | | Mouse 3 | |
| --- | --- | --- | --- | --- | --- | --- |
| Exons | Avg C_T_ | Std Dev | Avg C_T_ | Std Dev | Avg C_T_ | Std Dev |
| 1a/2a | 32.14 | 0.51 | 30.66 | 0.3 | 31.91 | 0.28 |
| 1a/2b | 25.77 | 0.08 | 25.14 | 0.04 | 24.91 | 0.14 |
|  |  |  |  |  |  |  |
| 22/23 | 22.04 | 0.06 | 21.31 | 0.09 | 21.15 | 0.03 |
| 22/24 | 30.09 | 0.32 | 29.84 | 0.1 | 29.22 | 0.03 |
| 22/26 | 20.98 | 0.04 | 20.59 | 0.03 | 20.51 | 0.01 |
|  |  |  |  |  |  |  |
| 29/31 | 22.33 | 0.12 | 22.45 | 0 | 21.89 | 0.03 |
| 30/31 | 27.78 | 0.13 | 27.6 | 0.26 | 28.13 | 0.1 |
|  |  |  |  |  |  |  |
| 26/32 | 29.43 | 0.26 | 28.51 | 0.1 | 28.94 | 0.13 |
| 29/32 | 28.01 | 0.2 | 27.04 | 0.06 | 27.29 | 0.1 |
| 31/32 | 20.65 | 0.06 | 19.96 | 0.02 | 19.96 | 0.01 |
|  |  |  |  |  |  |  |
| 39/40 | 23.03 | 0.02 | 22.56 | 0.01 | 22.24 | 0.04 |
| 39tr/40 | 30.44 | 0.4 | 31.11 | 0.56 | 32.25 | 0.16 |

Rows are divided into 5 sections representing different *Ank3* spliced variants that were detected during the initial screen. Rare alternative spliced junctions (<1%) are highlighted in gray rows. Exon 1a forms junctions with exons 2a or 2b thereby representing 100% of all possible exon 1a spliced junctions. Subsequent qt-PCR analysis demonstrated that the splice junction 1a/2a represents less that 1%, while the splice junction 1a/2b represents greater than 99% of spliced junctions.
